# Extensive Microsatellite Variation in Rice Induced by Introgression from Wild Rice (*Zizania latifolia* Griseb.)

**DOI:** 10.1371/journal.pone.0062317

**Published:** 2013-04-24

**Authors:** Zhenying Dong, Hongyan Wang, Yuzhu Dong, Yongming Wang, Wei Liu, Gaojian Miao, Xiuyun Lin, Daqing Wang, Bao Liu

**Affiliations:** 1 Key Laboratory of Molecular Epigenetics of MOE and Institute of Genetics and Cytology, Northeast Normal University, Changchun, China; 2 The State Key Laboratory of Plant Cell and Chromosomal Engineering, Institute of Genetics and Developmental Biology, Chinese Academy of Sciences, Beijing, China; 3 Faculty of Life Science, Liaoning University, Shenyang, China; 4 School of Life Sciences, Changchun Normal University, Changchun, China; University of Nottingham, United Kingdom

## Abstract

**Background:**

It is widely accepted that interspecific hybridization may induce genomic instability in the resultant hybrids. However, few studies have been performed on the genomic analysis of homoploid hybrids and introgression lines. We have reported previously that by introgressive hybridization, a set of introgression lines between rice (*Oryza sativa* L.) and wild rice (*Zizania latifolia* Griseb.) was successfully generated, and which have led to the release of several cultivars.

**Methodology:**

Using 96 microsatellite markers located in the nuclear and organelle genomes of rice, we investigated microsatellite stability in three typical introgression lines. Expression of a set of mismatch repair (MMR) genes and microsatellite-containing genes was also analyzed.

**Results/Conclusions:**

Compared with the recipient rice cultivar (Matsumae), 55 of the 96 microsatellite loci revealed variation in one or more of the introgression lines, and 58.2% of the altered alleles were shared by at least two lines, indicating that most of the alterations had occurred in the early stages of introgression before their further differentiation. 73.9% of the non-shared variations were detected only in one introgression line, i.e. RZ2. Sequence alignment showed that the variations included substitutions and indels that occurred both within the repeat tracts and in the flanking regions. Interestingly, expression of a set of MMR genes altered dramatically in the introgression lines relative to their rice parent, suggesting participation of the MMR system in the generation of microsatellite variants. Some of the altered microsatellite loci are concordant with changed expression of the genes harboring them, suggesting their possible *cis*-regulatory roles in controlling gene expression. Because these genes bear meaningful homology to known-functional proteins, we conclude that the introgression-induced extensive variation of microsatellites may have contributed to the novel phenotypes in the introgression lines.

## Introduction

Microsatellites, also called simple sequence repeats (SSRs), are tandemly repeated DNA motifs of 1–6 bp in length. They have been detected ubiquitously in the genomes of all organisms analyzed so far, and eukaryotic genomes generally contain much more microsatellites than prokaryotes [1]–[3]. Compared with single copy DNA sequences, microsatellites showed higher variability (about 10^−2^–10^−3^ per locus per gamete per generation), which leads to their high polymorphism in terms of repeat numbers within species and populations [4]. Microsatellite has been used widely as a useful and efficient DNA marker for multiple purposes in plants, including genome mapping, QTL tagging, association mapping, marker assisted selection, genetic diversity, cultivar identification, population genetics, and taxonomic/phylogenetic analysis [5]–[7]. This is largely due to salient properties of microsatellite, such as genome-wide coverage, hypervariability, codominant inheritance, reproducibility, chromosome-specificity and amenability to high throughput genotyping [7].

Several mechanisms have been suggested to explain the high mutation rate of microsatellites, including single-stranded DNA slippage, double stranded DNA recombination (unequal crossing over and gene conversion), mismatch/double strand break repair, and retrotransposition [7], [8]. Replication slippage was generally thought to be the main mechanism for microsatellite repeat length variation [9]. Slippage of DNA polymerase can cause transient dissociation of the template and the nascent strand during replication of the microsatellites. Due to the repetitive nature of the tract, the two DNA strands possibly re-associate out of register, leaving one or more unpaired repeats on either of the template or nascent strand, and results in unpaired repeat loops [10]. If the distortion caused by these unpaired bases is not removed from the newly synthesized strand by the DNA mismatch repair (MMR) system, the result will be a loss (if the unpaired bases are on the template strand) or a gain (if the unpaired bases are on the nascent strand) of one or more repeats [10], [11].

The MMR system is composed of proteins that are able to recognize mismatched DNA created during replication or recombination and ensure their correction. Inactivation of MMR genes could dramatically cause microsatellites instability by failing correcting replication errors [12]–[15]. In *Escherichia coli*, it was found that three MMR genes, *MutS*, *MutL* and *MutH*, are involved in this process. The MutS protein recognizes the mismatch, MutH introduces a nick in the target strand, and MutL mediates the interactions between MutH and MutS and stimulates the MutH associated endonuclease activity [16], [17]. MMR system is more complicated in eucaryotic organisms, several *MutS* and *MutL* homologues were identified in yeast, plant and animal genomes. In eukaryotes, MMR is undertaken by the MutS and MutL homologues (MSH and MLH). Both MSH and MLH polypeptides form MSH and MLH heterodimeric proteins, respectively, which act together to bind mismatched DNA and initiate repair. The MSH4–MSH5 heterodimer has only been reported to be involved in meiotic recombination [18], while the three remaining dimers are involved in both recombination and MMR. The MSH2–MSH6 heterodimer (MutSα) binds base mispairs and small insertion-deletion loop-outs [19], [20], The MSH2–MSH3 heterodimer (MutSβ) binds insertion/deletion loop-outs, while the plant specific MSH2–MSH7 heterodimer (MutSγ) binds base mispairs but not insertion-deletion loop-outs [21]. These heterodimers then recruit MLH proteins to initiate MMR. In human, microsatellite variation is often a hallmark of MMR gene deficiency and is used clinically to assess MMR proficiency in mammalian tumors [22]. In plants, microsatellite instability is also closely associated with MMR system. For example, Leonard et al. [13] found that stability of *Arabidopsis* genomic and transgenic DNA-repeat sequences was reduced by inactivation of AtMSH2 which is the constant component in MutSα, MutSβ and MutSγ. And the microsatellite instability can be increased up to 60-fold, in the RNA-interference (RNAi) lines [23].

Distribution of microsatellites within a given genome is not random [11]. In *Arabidopsis* and rice, the two sequenced model plant genomes of dicots and monocots, respectively, microsatellites are significantly enriched within the genic 5′ un-translated regions [24], indicating this kind of tandem repeats may have important functions. For example, computational scans of the genomes of *Arabidopsis* and *Brassica* showed that some microsatellites in 5′ un-translated regions were conserved and appeared to be ancient, and gene expression analysis showed that they may serve as regulatory elements involved in light and salicylic acid responses [25]. In other organisms, microsatellites in the 5′ un-translated regions were found to participate in regulation of transcription factor binding and gene expression [26], and specify modification in behavioral traits [27]. Microsatellites in introns were reported to affect gene transcription and splicing [22]. For example, (TCAT)n located in the first intron of the gene encoding tyrosine hydroxylase acts as a transcription regulatory element *in vitro* [28].

Interspecific hybridization has played an important role in plant evolution as well as a widely used method for introducing useful traits to crops [29]–[31]. Genetic analyses of hybrid segregating populations have shown that skewed segregation ratios are common [30], indicating genomic rearrangement or variation occurred as a result of the merge of divergent genomes. Studies from newly synthesized allopolyploids showed that wide hybridization and the concomitant whole genome duplication (WGD) had a profound impact on plant genome evolution. Extensive heritable genetic and epigenetic changes were found in the derived allopolyploids [32]–[35]. In contrast, few studies have been performed on the genomic analysis of homoploid hybrids. Even so, the experiments conducted in three sunflower species, *Helianthus annuus*, *H. petiolaris*, and *H. anomalus*, are especially revealing: *H. anomalus* is the putative diploid hybrid derivative form *H. annuus* and *H. petiolaris*. Rapid genomic rearrangement was detected in *H. anomalus*, which enabled its rapid ecological speciation [36], [37].

By the method of introgressive hybridization [38], we have successfully produced a set of introgression lines between rice (*Oryza sativa* L.) and the sexually incompatible wild rice (*Zizania latifolia* Griseb.), which have exhibited a wide range of novel phenotypes and led to the release of several rice cultivars [39]. The introgression lines are derived from a single “F1” plant, followed by repeated backcrossing with the rice parental cultivar Matsumae [39]. Using AFLP markers, we estimated less than 0.1% of *Zizania* genome DNA being introgressed into and still retained in these introgression lines [40]. Unexpected, however, both genetic and epigenetic variations were extensively detected in the introgression lines [40], [41]. Moreover, mobilization of several transposable elements was detected in the introgression lines [42]–[44]. These results suggest that the wide range of phenotypic variations in the introgression lines might be not a direct result of the integration of the *Zizania* DNA; instead, they were likely due to secondary genomic modifications triggered by *Zizania* DNA introgression, which is reminiscent of the findings in cultured animal cells in which random integration of foreign DNA has caused extensive epigenetic and transcriptome changes of the host genome [45]–[47].

In this study, we examined genomic insatiability of microsatellite loci in three representative rice-*Zizania* introgression lines, RZ1, RZ2 and RZ35. Our results reveal that introgression of a small amount of *Zizania* DNA has caused extensive and rapid sequence variation at microsatellite loci in the introgression lines, and the variation may have occurred rapidly during or immediately after introgression. We discuss possible role of the MMR system in regulating microsatellite stability, and possible contribution of microsatellite variation to the novel phenotypes in the introgression lines.

## Results

### Selection of Microsatellite Markers

**Figure 1:**
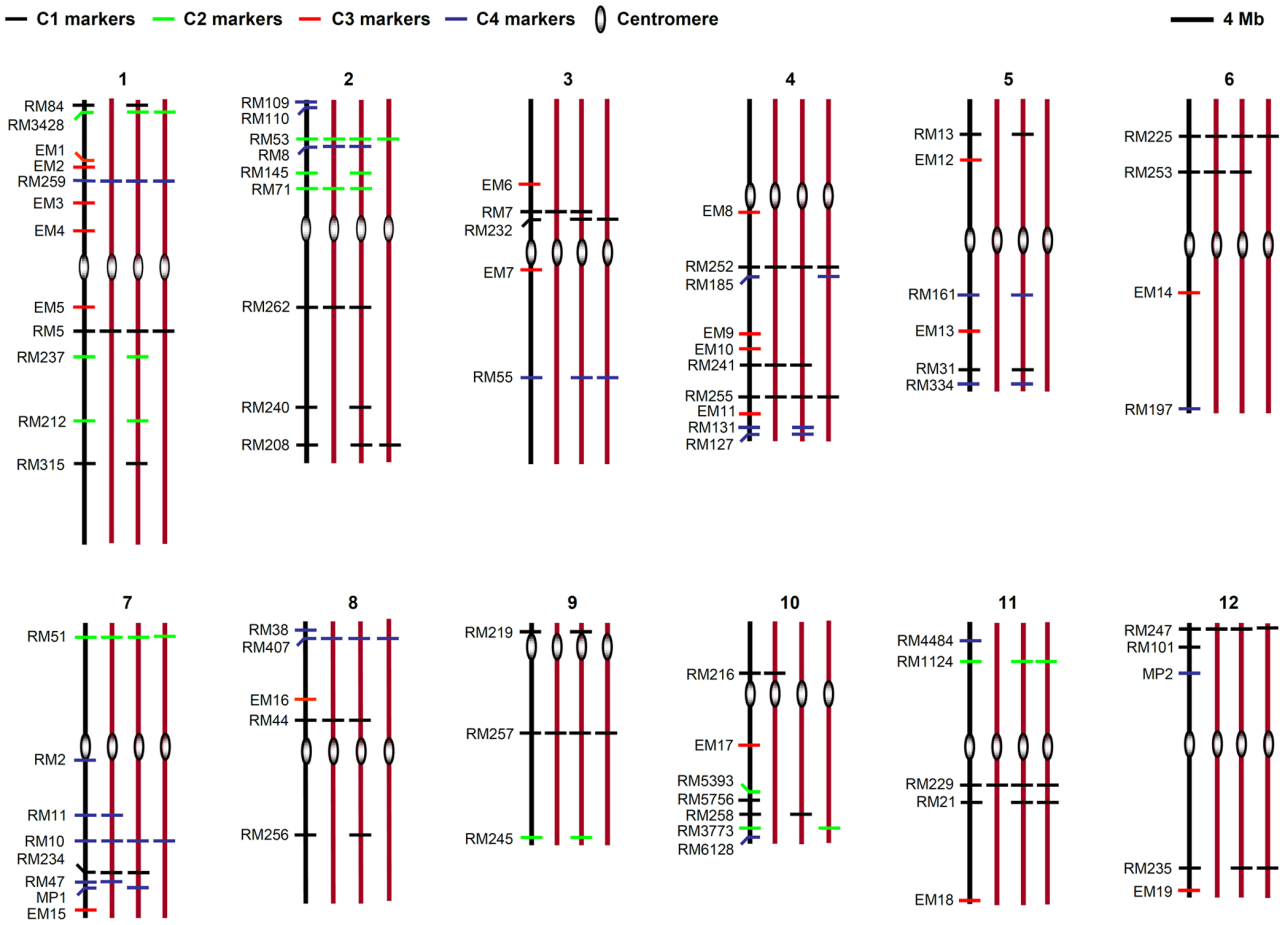
Distribution of the nuclear microsatellite markers in the 12 rice chromosomes. For each chromosome, the black vertical line represents Matsumae, and the three red vertical lines represent the introgression lines, RZ1, RZ2 and RZ35, respectively. Locations of the microsatellite markers were labeled by horizontal bars in Matsumae, while the horizontal bars in the three introgression lines denote microsatellites that showed polymorphism in the given introgression line(s). Different colors of the horizontal bars represent the different categories of microsatellite markers specified in the main text.

Nighty-six microsatellite markers mapping to the rice nuclear and organelle genomes were used to examine the genetic variation in the three introgression lines (RZ1, RZ2 and RZ35) by comparison with their rice and wild rice parents, Matsumae and *Z. Latifolia*. Seventy-nine of the used microsatellite markers located in the rice nuclear genome (Figure 1, Table S1). Of these, one marker every 4.8 Mb sequence on average was estimated based on the published rice genome data (International Rice Genome Sequencing Project, IRGSP, 2005). The study of Grover et al. [48] detected 134,822 microsatellite loci and 132,825 microsatellite loci in *japonica* and *indica* genome, respectively. Accordingly, our markers cover about 0.07% of the microsatellite loci in the rice genome. These markers were further assigned to all the 12 rice chromosomes, with chromosome 1 contained the largest number of markers, and chromosomes 6 and 9 had the least number of markers (Figure 1, Table S1). Depending on the genome data from IRGSP (2005), one marker per 3.3 Mb sequences in rice chromosome 1 was estimated. While in the remaining 11 chromosomes, one marker per 4.1, 6.2, 3.6, 5.6, 7.9, 4.3, 5.7, 10.2, 3.4, 6.2 or 5.6 Mb sequences was deduced, respectively. Depending on the location relative to genes, the 79 nuclear markers can be divided into four categories (Figure 1, Table S1). Specifically, category one (C1) contained 28 markers that are located in nuclear intergenic regions. Except for RM5756 (examining AGA repeats), all the markers in this category were used to detect dinucleotide-repeat microsatellites. Category two (C2) contained 11 markers located in intronic regions, which included one trinucleotide-repeat markers (RM71) and 10 dinucleotide-repeat markers. Category three (C3) contained 19 markers located in gene-coding regions and thus are exonic markers, which were developed in this study. Fifteen (78.9%) of the markers were to examine tri-nucleotide repeats, four (21.1%) were to examine dinucleotide-repeats, and one (5%) were to examine mononucleotide-repeats. Category four (C4) contained 21 markers residing at gene promoter regions, with two markers (MP1, MP2) were newly developed in this study. We defined this category of markers only when the microsatellite was no more than 1 kb upstream the gene start codon (ATG). Fifteen (71%) of the markers were to detect dinucleotide-repeats and six (29%) were to detect trinucleotide-repeats.

Of the 10 rice chloroplast and seven mitochondrial located microsatellite loci investigated in this study, only three categories of markers (C1, C2 and C3) were identified (Table S1), and all these organellar DNA markers were to examine mononucleotide-repeat microsatellite loci (Table S1).

### Extensive Variation was Detected in all Three Introgression Lines by the Microsatellite Markers

**Figure 2:**
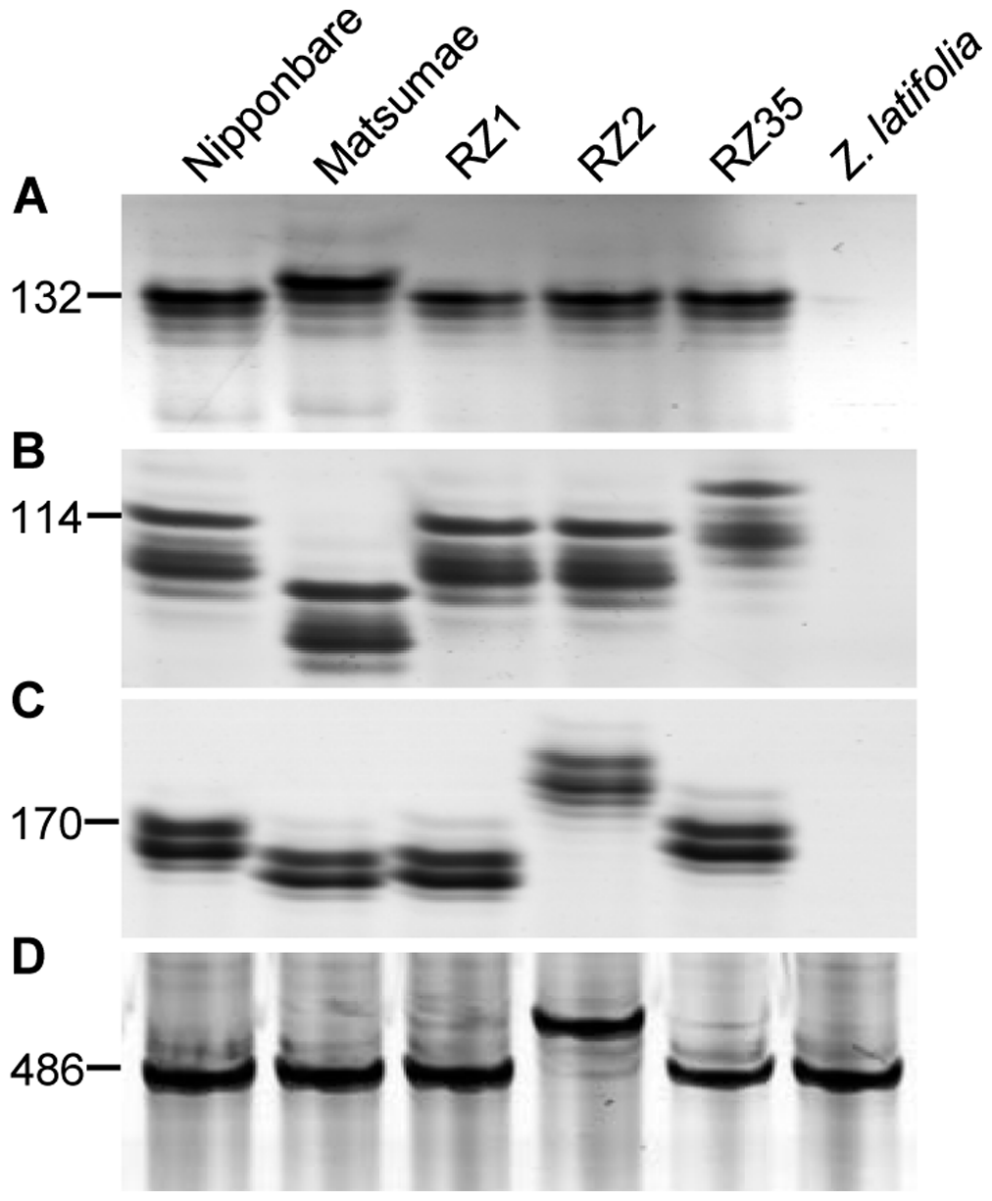
PCR amplification patterns of the microsatellite markers between the introgression lines and their parental lines. (**A**) Marker RM225; (**B**) Marker RM5; (**C**) Marker RM208; (**D**) Marker RMt9. lengths (bp) of the PCR products from Nipponbare were indicated.

Using primers for the above microsatellite markers, PCR experiments were performed using DNA templates from the three introgression lines and their parental cultivars Matsumae and *Z. latifolia*. To evaluate specificity of the markers, the standard laboratory *japonica* rice cultivar Nipponbare was used as a positive control. Similar bands with Nipponbare were produced from the introgression lines and Matsumae in all 96 microsatellite loci (e.g., Figure 2). It is interesting to note that, when using DNA of *Z. latifolia* as template, no PCR product was amplified from any of the primer pairs for the nuclear markers (e.g., Figure 2A, B and C), indicating significant divergence at the microsatellite loci between rice and *Zizania* belonging to two distinct genera.

By comparison of amplification patterns between the introgression lines and their rice parent Matsumae, length of the amplified bands changed in multiple microsatellite loci. In total, 55 (57.3%) of the analyzed microsatellite loci detected length variation in the introgression lines compared with Matsumae (Table. 1). Twenty-three (24.0%) of the microsatellite markers detected variation in RZ1 *vs.* Matsumae and RZ35 *vs.* Matsumae respectively, while 49 (51%) of the markers detected variation in RZ2 *vs.* Matsumae (Table. 1). For the nuclear loci, RZ1, RZ2 and RZ35 detected sequence length variations by 22, 44, and 21 microsatellite markers respectively. As a result, 49 loci detected polymorphisms in the three introgression lines in total. Of the four categories of microsatellite markers, 26 (92.9%) of the C1 markers, 10 (90.9%) of the C2 markers and 13 (61.9%) of the C4 markers, respectively, showed polymorphism in at least one introgression line (Table 1). All these three categories of microsatellite markers detected much higher mutational rates in RZ2 than in RZ1 and RZ35 (Table 1). Interestingly, no polymorphism was detected by the 19 C3 microsatellite markers in any of the introgression lines (Table 1).

**Table 1:** Classification of the 96 microsatellite markers used in this study and their variation frequencies in the introgression lines.

Markers			Variation			
Location	Category	Number	RZ1	RZ2	RZ35	Total
Nuclear	C1	28	14	25	11	26 (92.9%)
	C2	11	5	9	4	10 (90.9%)
	C3	19	0	0	0	0 (0%)
	C4	21	3	10	6	13 (61.9%)
Chloroplast	C1	5	1	2	1	3 (60.0%)
	C2	2	0	1	0	1 (50.0)
	C3	3	0	1	1	1 (33.3%)
Mitochondrial	C1	4	0	0	0	0 (0%)
	C2	1	0	0	0	0 (0%)
	C3	2	0	1	0	1 (50.0%)
Total		96	23 (24.0%)	49 (52.6%)	23 (24.0%)	55 (57.3%)

Using the 10 chloroplast and seven mitochondrial microsatellite markers, similar bands with Nipponbare were amplified from Matsumae, introgression lines and *Z. latifolia* (Figure 2D). The variation in the introgression lines showed similar patterns with that in nuclear loci, that is, RZ2 showed the highest mutation rate among the three introgression lines (Table 1). The largest difference with the results from nuclear microsatellite loci is the polymorphism in exonic locus (C3) of organellar genomes, i.e., RZ2 and RZ35 showed variations in RCt7 locus, and RZ2 showed variation at locus RMt9 (Table 1).

Although the three introgression lines were derived from the same F1 hybrid plant, both shared and non-shared bands in the polymorphic loci were discovered (Figure 1). Three types of polymorphisms were identified, type I referred to the loci that all the introgression lines shared the same polymorphism relative to their rice parental cultivar Matsumae (Figure 2A), type II referred to the loci that two of the three introgressants shared the same polymorphism relative to Matsumae (Figure 2B), and type III referred to the loci that each of the introgressant showed distinct polymorphism (Figure 2C, D). For the nuclear microsatellite loci, 4.1% (2/49) of the polymorphic loci belonged to type I, 55.1% (27/49) of the polymorphic loci belonged to type II, and 40.8% (20/49) of the polymorphic loci belonged to type III (Table 2). It’s worthy to note that 14 of the type III polymorphisms were due to the sequence variation happened in RZ2 (Table 2). For the organellar microsatellite loci, only type II and type III polymorphisms were found in the introgression lines, and all the type III polymorphisms were caused by the sequence variation in RZ2 (Table 2). As the result, the shared variation (type I and II) made up 58.2% (32/55) of all the polymorphisms in the introgression lines (Table 2). The no-shared variation (type III) took 41.8% (23/55) of the polymorphisms, and 73.9% (17/23) of the variation was only detected in RZ2.

**Table 2:** Microsatellite Variation patterns in the introgression lines.

Location	No. Totalloci	No. Polymorphicloci	Variation pattern		
			I	II	III
Nuclear	79	49	2	27	14*/6
organellar	17	6	0	3	3*
Total	96	55	2	30	17*/6

* Indictes the variations happened only in RZ2.

**Figure 3:**
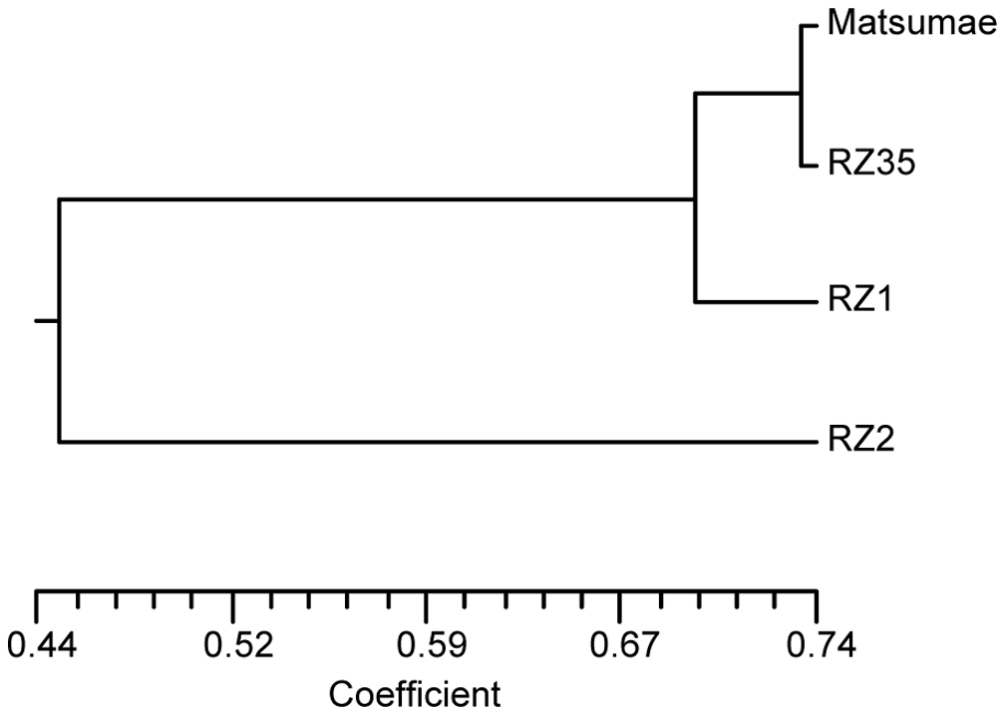
A UPGMA dendrogram to show the overall genomic differentiation of the introgression lines from their rice parental cultivar. The Dendrogram was generated by NTSYS software using the Jaccard’s coefficient of similarity calculated on the microsatellite markers.

To analyze the genetic divergence between the introgression lines and Matsumae, we made cluster analysis based on the alleles generated by the microsatellite markers, and a dendrogram was generated as shown in Figure 3. It was estimated that the genetic distances between Matsumae and RZ1, RZ2, or RZ5 was 0.31, 0.77 or 0.27, respectively, pointing to substantial genome-wide differentiation of the introgression lines from their parental rice genotype.

### Stable Inheritance of the Altered Microsatellites in the Introgression Lines

**Figure 4:**
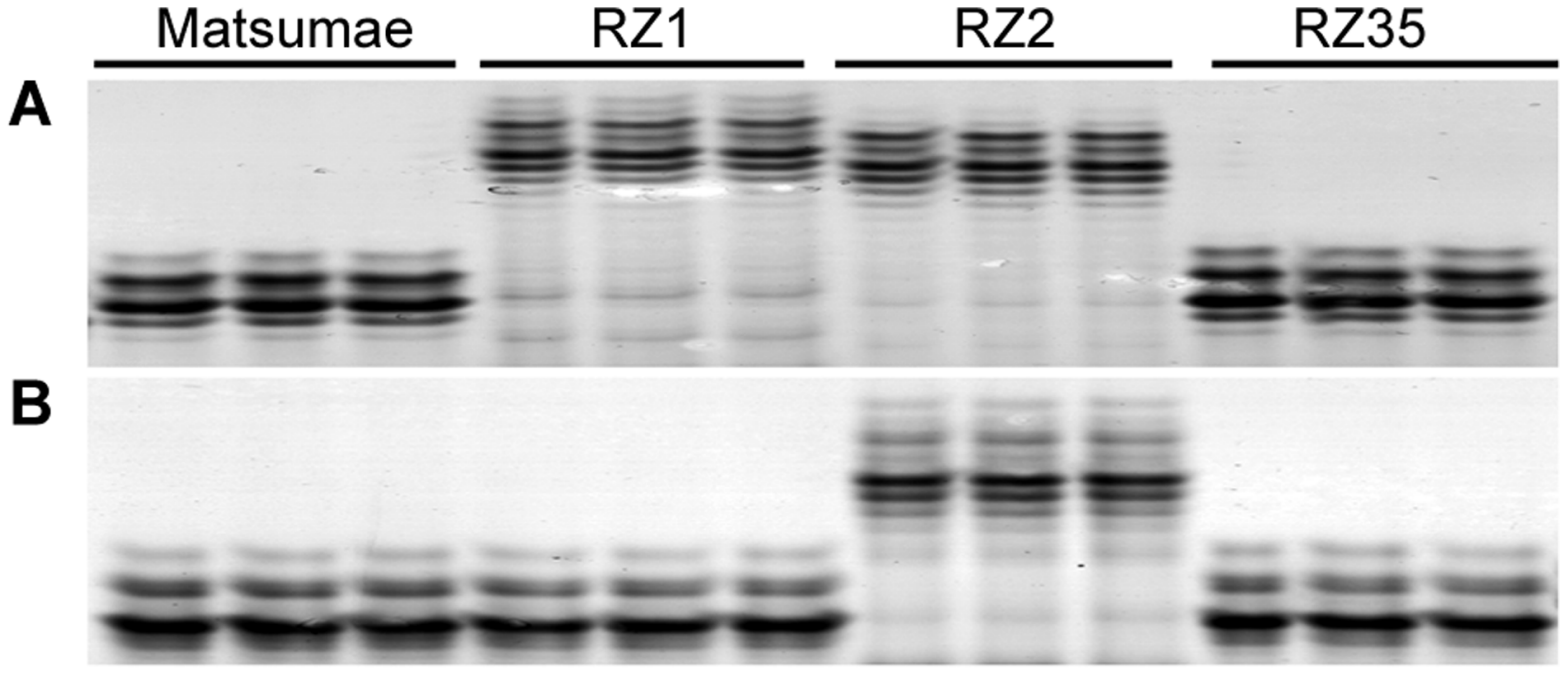
Examples of genomic stability in Matsumae, RZ1, RZ2 and RZ35. (**A**) Marker RM234; (**B**) Marker RM258.

To investigate whether the altered microsatellites in the introgression lines were mitotically stable and meiotically heritable, genomic DNA from three randomly selected individual plants taken from each of the three successive selfed generations (9^th^) were used as PCR templates, and amplified with 10 randomly selected microsatellite marker primers. Results showed that complete uniformity in band patterns among the individuals for a given line was detected both within and between the selfed generations (Figure 4), indicating stable mitotic perpetuation and meiotic inheritance of the altered band patterns in the introgression lines.

### Nature of the Microsatellite Variations at the Primary DNA Sequence Level

**Figure 5:**
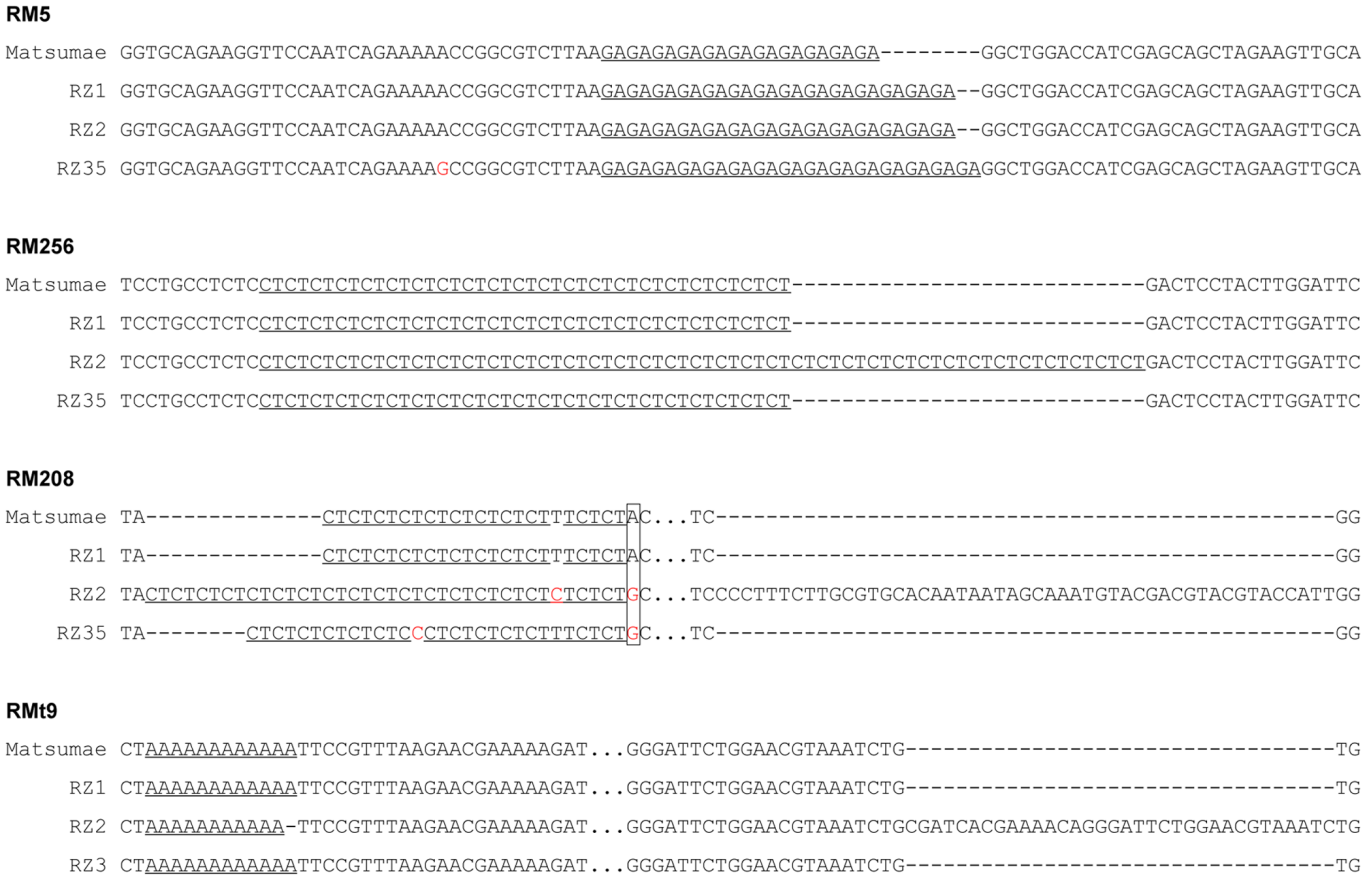
Examples of the sequence variations happened in microsatellite loci of the introgression lines. The repeat sequences were underlined, and the indels were indicated by dashed lines. The substitution sites were labeled in red, while the substitutions shared by introgression lines were framed. The dots represented the un-displayed nucleotides.

To gain further insights into the nature of the variations, 46 polymorphic bands from the introgression lines at 30 microsatellite loci (from 25 of nuclear and 5 of organellar genomic loci) were recovered and sequenced. At the same time, 30 bands at the corresponding loci from Matsumae were also sequenced. In total, 7,394 nucleotides were obtained from three introgression lines. Multiple alignments indicated that changes of microsatellite repeat numbers were detected at each locus (e.g., Figure 5). And coincide with the results revealed by electrophoresis, mutational frequency in RZ2 was highest among the three introgression lines. The maximal microsatellite variation occurred at the RM256 locus of RZ2. In this locus, Matsumae was detected as (CT)_21_, but it was (CT)_35_ in RZ2 (Figure 5). The minimal change was also detected in RZ2, for the repeat tract of RMt9, in which only one repeat unit difference between RZ2 and Matsumae (Figure 5). Apart from the changes in repeat numbers, base substitutions within the repeat tracts also occurred. For example, there was an imperfect repeat (CT)_12_TT(CT)_2_ at the RM208 locus of Matsumae, but the motifs were (CT)_6_CC(CT)_5_TT(CT)_2_ in RZ35 and (CT)_19_ in RZ2, respectively (Figure 5), indicating thymine to cytosine mutation occurred in both RZ35 and RZ2.

Sequence variation in the flanking regions of microsatellites was another dramatic characteristic between the introgression lines and Matsumae. And not like the short sequence insertions or deletions which mainly occurred in the repeat regions, long fragment insertions were also detected in the flanking regions. For example, RZ2 contained five more dinucleotide CT repeats relative to Matsumae at locus RM208, while a 43 bp sequence inserted into the flanking region of the microsatellite (Figure 5). As a result, the sequence of RZ2 was 53 bp longer than that of Matsumae at this locus. The only sequence length polymorphism detected in mitochondrial genome microsatellite loci was at RMt9 of RZ2 (Figure 2D). Sequencing results demonstrated a single nucleotide (A) was deleted in the repeat tracts, but 37 bp sequences were inserted into the repeat flanking region of RZ2 (Figure 5). Consistent with the results from gel analysis, both shared and nonshared variations were detected by sequence alignments (Figure 5). We noticed that, in some loci, nucleotide substitutions and/or indels in flanking regions were also shared by the introgression lines, while the repeat numbers of microsatellites were different from each other (Figure 5), indicating the repeats are more unstable than the flanking sequences and have been undergoing additional changes.

Among the 7,394 bp sequences of the introgression lines, 1,233 bp (16.7%) were in the repeat tracts and 6,161 bp (83.3%) were in the flanking regions. To compare the mutational frequency, nucleotide substitution, insertion and deletion were compared between these two kinds of regions (Table 3). For nucleotide substitution, relative higher mutational rate was only observed in the microsatellite flanking sequences which acquired from C1 loci (Table 3). Indels were the main mutational mechanism in both kinds of regions, and although fewer nucleotides were obtained from microsatellite repeat tracts compared with the flanking regions, more mutational sites and nucleotides were observed within the repeats (Table 3). But variation in C3 loci was an exception, primarily because the only sequenced polymorphic band (from RZ2 at locus RMt9) contained a long fragment insertion in the flanking region (Figure 5).

**Table 3:** Comparison of microsatellite variation in the repeat tracts and the flanking regions in the introgression lines at the nucleotide sequence level.

category^a^	No. sequenced bands in the introgression lines	Variation Location	Sequenced nucleotides(bp)	No. Nucleotidesubstitution	Indel			
					Deletion		Insertion	
					Site	Length (bp)	Site	Length (bp)
C1	10	Flanks	1,551	13	1	2	3	51
		Repeats	307	2	4	21	6	74
C2	8	Flanks	1,164	6	2	3	1	2
		Repeats	340	0	6	61	2	19
C3	1	Flanks	475	0	0	0	1	37
		Repeats	11	0	1	1	0	0
C4	27	Flanks	2,971	3	2	4	1	1
		Repeats	575	3	14	79	6	52
Total			7,394	27	29	170	19	199

a Both nuclear and organellar genomic microsatellite markers were combined together and classified into four categories (C1 to C4) depending on their location relative genes.

### Altered Expression of the MMR Genes in the Introgression Lines

**Figure 6:**
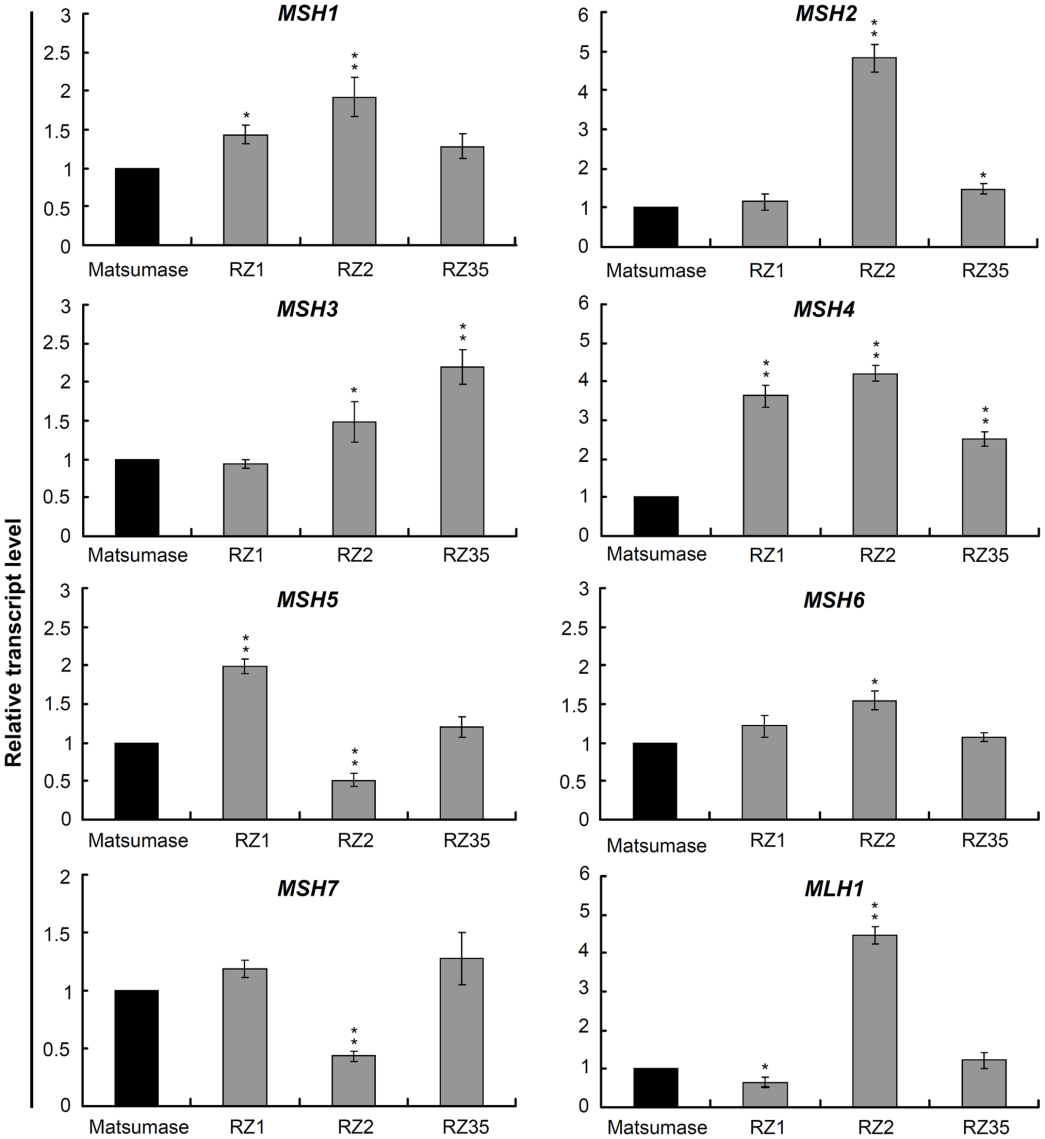
Evaluation of the relative transcript levels of the eight MMR genes in Matsumae, RZ1, RZ2 and RZ35, by quantitative RT-PCR. Total RNA samples were prepared from the leaves at three-leaf stage, and were used for cDNA synthesis and the subsequent quantitative PCR with gene specific primers. Each data point was calculated with the results of three technical repeats.

Expression of eight MMR genes, including seven homologous to *MutS* (*MSH1*-*7*), and one (*MLH1*) homologous to *MutL*, were investigated using real-time qRT-PCR in Matsumae and the introgression lines. The steady-state transcript abundance of all the eight genes differed substantially from each other in the three introgression lines and from their rice parent Matsumae (Figure 6). Five genes, *MSH1*, *MSH2*, *MSH3*, *MSH4* and *MSH6*, only detected up-regulation in the introgression lines *vs.* Matsumae, while both up- and down-regulation were detected for the remaining three genes in the introgression lines (Figure 6). For RZ1, two genes (*MSH4* and *MSH5*) were up-regulated significantly (P<0.01) relative to Matsumae. For RZ2, four genes (*MSH1*, *MSH2*, *MSH4*, and *MLH1*) were up-regulated significantly, and two genes (*MSH5* and *MSH7*) were down-regulated significantly (P<0.01), relative to Matsumae. For RZ35, only *MSH3* and *MSH4* detected significant up-regulation (P<0.01). Combining results of all three introgression lines, only *MSH4* showed significant up-regulation compared with Matsumae (Figure 6). Combining the eight MMR genes, three (*MSH2*, *MSH4* and *MLH1*) exhibited a relatively high increase of the transcript level relative to Matsumae than others, and the transcripts of *MSH2* and *MLH1* only showed significant accumulation in RZ2 (Figure 6).

### Expression of Microsatellite-associated Genes

**Figure 7:**
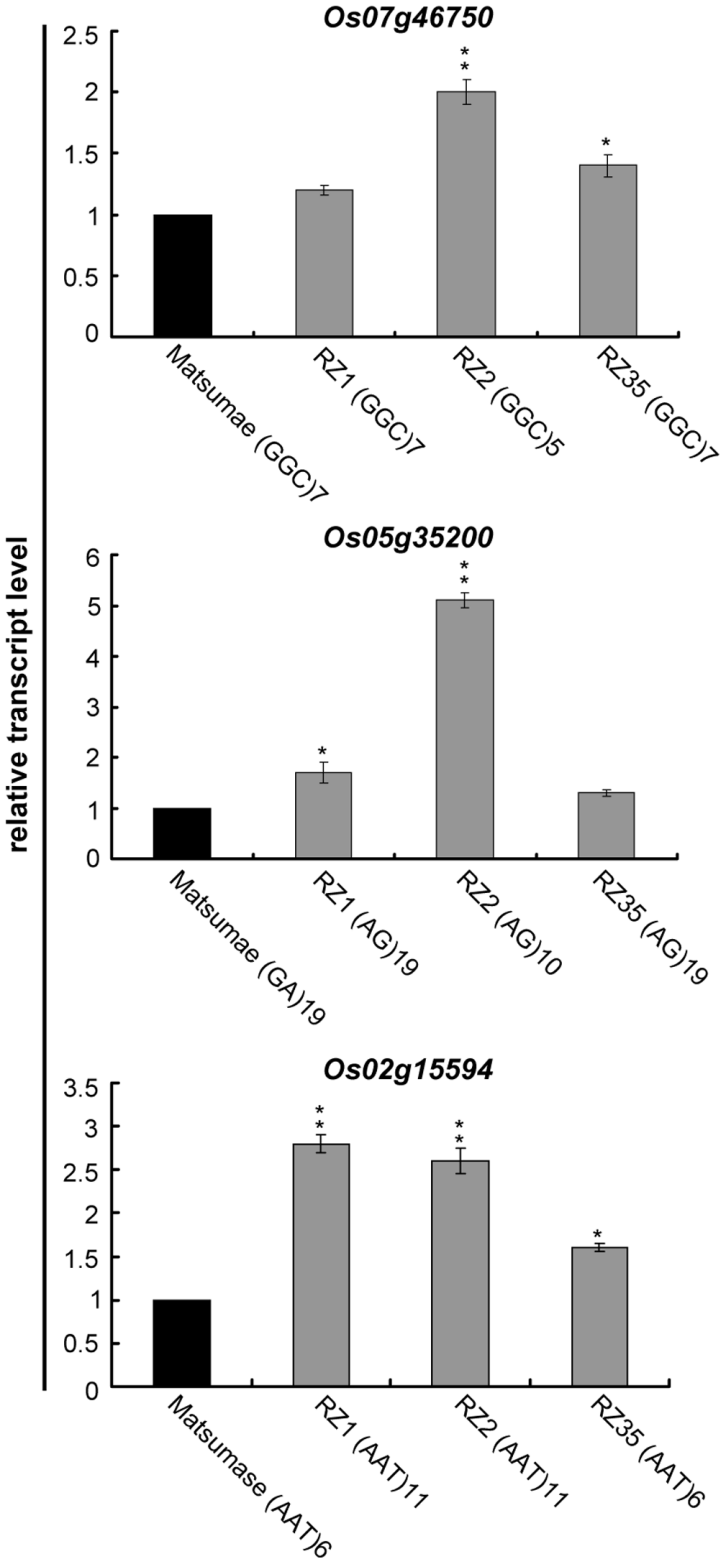
Evaluation of the relative transcript levels of the three microsatellite possibly regulated genes in Matsumae, RZ1, RZ2 and RZ35, by quantitative RT-PCR. (**A**) Transcript levels of *Os07g46750*; (**B**) Transcript levels of *Os05g35200*; (**C**) Transcript levels of *Os02g15594*.

As shown by many studies, microsatellite repeat motifs located in the *cis*-regulatory regions can regulate gene transcription [26], [27]. To examine whether microsatellite variation had an effect on gene expression in the introgression lines, two sets of genes were selected for gene expression analysis. The first set (Set 1) refers to those that contained microsatellites in the promoter regions, and the microsatellites were detected variation in introgression lines. The second set (Set 2) refers to those that contained microsatellites in the introns, and the microsatellites were detected variation in the introgression lines. Thirteen genes in Set 1 and 10 genes in Set 2 were examined by real-time qPCR. Results showed that expression of 20 genes in the introgression lines showed no significant alteration compared with Matsumae, but transcripts of two genes of Set 1 (*Os07g46750* at MP1 locus and *Os05g35200* at RM161 locus) and one gene of Set 2 (*Os02g15594* at RM71 locus) showed substantial difference in the introgression lines (Figure 7). At the MP1 and RM161 loci, the repeat number in RZ2 was reduced (Figure 7), while expression of *Os07g46750* and *Os05g35200* was significantly up-regulated in RZ2 (*P*<0.01) (Figure 7). At the RM71 locus, the repeat number was increased in RZ1 and RZ2, while expression of *Os02g15594* in these two introgression lines was significantly up-regulated (P<0.01) (Figure 7).

To further examine if these microsatellites have a common effect on gene expression, we collected a set of rice cultivars that contained the same alleles as Matsumae or the introgression lines at the MP1, RM71 and RM161 loci, respectively. Results showed that the gene expression levels showed statistically significant deviation between the two groups of rice cultivars (Figure S1). Specifically, the three genes in rice cultivars containing the same numbers of simple repeats with Matsumae all showed relatively low expression levels, while in rice cultivars containing the same numbers of simple repeats as introgression line (RZ1 or RZ2) at these genes, all showed higher expression levels (Figure S1). These data suggest that alterations in gene expression patterns in different genotypes may be conferred via microsatellite variation resided in the *cis*-regulatory regions of the genes.

## Discussion

Hybridization between genetically differentiated plants has played a pervasive role in genome evolution [49]–[51]. Currently, studies on hybrid genome evolution were mainly focused on allopolyploids [40], primarily because homoploid hybrid speciation is rarely detected compared with allopolyploidy [51], [52]. We have reported that the homoploid hybrid derivatives from hybridization between rice and *Z. Latifolia*, i.e., the introgression lines, exhibited heritable and novel phenotypes compared with their rice parental cultivar Matsumae [39], [43]. Although <0.1% of *Z. Latifolia* DNA introgression was detected [40], extensive genetic and epigenetic instability including mobilization of otherwise quiescent transposable elements were extensively detected in the introgression lines [40]–[44]. To extend the earlier findings, we have examined genetic stability at the microsatellite loci of introgression lines in this study, and new results and conclusions are obtained as discussed below.

### Minute *Z. latifolia* DNA Introgression had a Potent Effect and Induced Extensive Microsatellite Variation in the Rice-*Zizania* Introgression Lines

Using 96 microsatellite markers, the amplification patterns were examined in Matsumae, *Z. latifolia*, and the three introgression lines. Interestingly, no PCR product was amplified from *Z. latifolia* using the 79 nuclear genome markers (Figure 2A, B and C). This indicated that the corresponding microsatellite loci are not present in the *Z. latifolia* genome, consistent with the remote phylogenetic relationship between rice (*Oryza*) and *Zizania* as two genera. As such, the microsatellite alleles detected in the introgression lines can only be from their rice parent cv. Matsumae. In contrast, the 17 organellar DNA markers were found to present in both Matsumae and *Z. latifolia* (Figure 2D). However, *Z. latifolia* was the paternal parent [39], [43], therefore the organelle marker alleles in the introgression lines were also exclusively contributed by rice (cv. Matsumae). Taken together, all variations at the microsatellite loci detected in the introgression lines should have occurred *de novo* as a result of introgressive hybridization.

We found that the nuclear microsatellite loci showed strikingly variable mutational frequencies depending on their locations relative to genes, with microsatellites of the three kinds of non-coding regions (C1, C2 and C4) mutated extensively, while the sequences at C3 loci which resided in coding regions are largely stable (Table 1). This result is different from our previous study showing that genomic variations at randomly sampled loci across the genome by the AFLP markers were represented by non-coding and coding regions at similar frequencies [40]. The contrasting results of genetic variation frequencies detected in the introgression lines between the two kinds of markers, AFLP and SSR, may suggest that variations of microsatellites in gene coding regions is more functionally constrained. Microsatellite variation in gene coding sequences may lead to generation of premature stop codons, frame-shift mutations (e.g., repeat number variation in dinucleotide repeats) or disruption of functional motifs. In human, for example, SSR expansion mutations have caused neurological diseases, and the largest class of these diseases results from the expansion of coding CAG repeats that are translated into extended (Gln)n tracts within the corresponding proteins [22].

Surprisingly, microsatellite variation was also detected in the chloroplast and mitochondrial genomes in the introgression lines, including those occurred in gene coding regions. This raises an interesting issue and suggesting that hybridization may also impact genetic stability of cytoplasmic genomes that are only inherited from the maternal parent. Mechanisms for this intriguing observation warrant further investigations.

### Most Microsatellite Variations in the Introgression Lines Occurred at Early Developmental Stages in the F1 Hybrid, and Followed by Stabilization

Of the 96 investigated microsatellite loci, 55 (57.3%) detected variation in at least one of the three introgression lines. Of these variable loci, 32 (58.2%) were shared by two or all three introgression lines (Table 2). This suggests that most of the microsatellite variations occurred before differentiation of the three introgression lines from the common F1 hybrid individual of rice (cv. Matsumae) and *Zizania* [43]. Investigation from a set of individual plants indicated stable mitotic perpetuation as well as meiotic inheritance of the altered banding patterns in the introgression lines (Figure 4). Thus, our results suggest that most if not all of the microsatellite variations that were detected in the introgression lines had occurred in the somatic primordial cells of the F1 hybrid before differentiation of gametal cells; thereafter, the variant alleles were largely stabilized.

### Variations at Both Repeat Tracts and their Flanking Regions Underlie the Microsatellite Variations in the Introgression Lines

At the primary sequence level, changes in the repeat number were detected in all of the sequenced microsatellite loci, and the variation scope ranged from one to 14 repeat units. In contrast, base substitutions were rarely detected within the repeat tracts, consistent with the suggestion that substitutions in microsatellite repeats may decrease the mutation rate of microsatellite loci [53]. Interestingly, variations that converted an imperfect microsatellite to a perfect one were found in the introgression lines, especially in RZ2 (e.g., RM208), further indicating the diverse nature of microsatellite variations in the introgression lines.

Nucleotide substitutions, deletions and insertions were frequently detected in the microsatellite flanking regions, indicating that variations in both repeat tracts and their flanking regions have contributed to the microsatellite polymorphism in the introgression lines. This is in line with the emerging opinion that due to their physical connectivity, co-evolution of microsatellites and their flanking regions is a rule [54], [55].

Consistent with the above discussion, sharing of the variations by the introgression lines was also detected by detailed sequence analysis. For example, the same repeat number variation was observed in all three introgression lines at locus RM5, while the same substitution was detected in RZ2 and RZ35 flanking the repeat tract at locus RM208 (Figure 5). These results further support the conclusion that variations in microsatellite loci detected in the introgression lines occurred in the early stage before differentiation of the introgression lines.

### Dysregulation of the Mismatch Repair (MMR) Genes Likely Contributed to the Microsatellite Variation in the Introgression Lines

Slipped-strand mispairing during DNA replication is considered as the leading mutational mechanism for microsatellite instability [9]. Conceivably, most of the mutations occurred due to this cause would have been corrected by the normal function of the cellular MMR system [56]. It was reported that MMR-deficient cells or organisms exhibited a significant increase in frequency of point mutations and microsatellite instability [57], [58]. The extensiveness of microsatellite variation in the introgression lines promoted us to assess expression of the MMR genes in the introgression lines relative to their rice parental cultivar. Indeed we found that the steady-state transcript abundance of all the eight assayed MMR genes differed substantially from each other in the three introgression lines as well as from their rice parental cultivar. Among these, five genes, *MSH1*, *MSH2*, *MSH3*, *MSH4* and *MSH6*, showed up-regulation in the introgression lines relative to Matsumae, while the remaining three genes showed either up- or down-regulation in the introgression lines. Based on these results, we suspect that in the F1 hybrid of rice and *Z. latifolia*, replication slippage likely occurred widely, and the up-regulation of most of these MMR genes to produce more functional proteins was probably a response to these events. As to why the up- or down-regulations of the MMR genes became stabilized across the selfed generations in the introgression lines remains unknown. However, one conceivable reason is that the *cis* and/or *trans* factors of the MMR genes themselves have been irreversibly modified as a results of the widely occurred genetic and/or epigenetic variations in the introgression lines [40], [41].

### Possible Functional Consequence of the Microsatellite Variation in the Introgression Lines

Variation of microsatellites residing at gene coding regions may cause frame-shift mutation and alter protein function, while those at noncoding regions may serve as *cis*-regulatory elements [25]–[27]. In plants, no direct evidence is yet available demonstrating a causal role of microsatellites in regulating gene expression. By analyzing 13 and 10 genes which contained microsatellites in promoters and introns, respectively, our results showed that most microsatellites had no apparent role in gene regulation, but the expression of three genes, *Os02g15594*, *Os05g35200* and *Os07g46750*, were found associated with microsatellite variation.

*Os02g15594* encodes a protein phosphatase. Protein phosphorylation plays a fundamental role in modifying protein functions, and this kind of proteins are involved in a number of signaling pathways triggered by abiotic stresses, and also play a roles in plant development [59]. *Os05g35200* encodes a glycoysltransferase. Glycoysltransferases play a key role in the biosynthesis of the cell wall components, and they catalyze the glycosylation of a range of diverse molecules involved in biological processes such as development, signaling, and defence [60]. *Os07g46750* encodes an elongation factor, which plays a central role in the elongation step of protein biosynthesis [61]. Thus, it is reasonable to conclude that the heritable alteration in the expression of these genes in the introgression lines likely have contributed to the novel phenotypes seen in these lines.

## Materials and Methods

### Plant Materials

Three introgression lines (RZ1, RZ2 and RZ35) derived from a cross between rice (cv. Matsumae) and a local accession of wild rice (*Z. latifolia* Griseb.). The hybridization approach used to produce these lines and their novel phenotypes relative to the rice parental line (Matsumae) were reported previously [39], [43]. All introgression lines and their rice parent were maintained by strictly controlled selfing under normal growing conditions. Genomic DNA was isolated from expanded leaves of individual plants by a modified CTAB method and purified by phenol extractions.

### Microsatellite Marker Development and Analysis

Nighty-six rice microsatellite markers were selected and used in this study. Of the 79 nuclear genome microsatellite markers, 58 were developed by McCouch et al. [62], and the remaining 21 markers were newly developed in this study. Primers were designed by the software primer premier 5 (PREMIER Biosoft International, Palo Alto, USA). After a Blast N analysis against the database of the standard laboratory cultivar Nipponbare genome (http://rice.plantbiology.msu.edu/), all the nuclear genomic markers were assigned to 12 rice chromosomes (Figure 1, Table S1). Of the 17 organellar genome specific markers, 10 located in chloroplast genome and seven located in mitochondrial genome. The primers for these markers were according to Hashimoto et al. [63], and Nishikawa et al. [64] respectively.

DNA from Matsumae, the three introgression lines and *Zizania* were used as templates to detect microsatellite polymorphism. PCR was carried out in a 25 µl volume containing 50 ng genomic DNA, 10 mM dNTPs, 5 pmol of each primer, and 0.2 U high-fidelity DNA polymerase (TaKaRa, Tokyo, Japan). The cycling parameters were: 94°C for 5 min, followed by 33 cycles of 94°C for 30 s, 60 to 62.5°C for 30 s and 72°C for 1 min, and a final extension at 72°C for 5 min. PCR products were separated in 5% denaturing polyacrylamide sequencing gels, the fragments were visualized by silver staining.

### Assessing Similarity Relationships among Matsumae and Introgression Lines

The amplified alleles by the 96 molecular markers were scored as present (1) or absent (0) for each locus. Cluster analysis was performed using the computer package NTSYS-PC version 2.01 [65]. Similarities between genotypes were estimated using Jaccard’s coefficient [66]. Cluster analysis was conducted based on similarity estimates using the unweighted pair group method on arithmetic averages (UPGMA) [67].

### Sequence Recovery and Multiple Alignments

To investigate the nature of length variation within specific loci, bands that showed polymorphism in the silver-stained denaturing polyacrylamide gels were eluted and re-amplified with the same primer combinations in the original microsatellite amplifications. The PCR fragments of the expected size were recovered from agarose gels, followed by cloning into the pGEM-T Easy vector (TaKaRa, Tokyo, Japan) and DNA sequencing. Three separate clones derived from independent PCR amplifications and cloning trials were sequenced for constructing the final nucleotide sequence of each amplicon. The accession numbers and the amplified sequences of the sequenced loci were provided in Table S2. Multiple alignments were performed using the ClustalW program [68].

### Reverse Transcription and Real-Time PCR

The leaf tissue from Matsumae and the three introgression lines were harvested at the three-leaf stage plants for RNA extraction. Total RNA was extracted by Trizol Reagent (Invitrogen, Carlsbad, USA), and the reverse transcription (RT) reaction was performed using a RT system (Invitrogen, Carlsbad, USA) following the manufacturer’s protocol. Primers used for quantitative RT-PCR were designed by the primer premier 5 (PREMIER Biosoft International, Palo Alto, USA). The primer sequences are given in Table S3 (primers for rice *β-actin* genes, eight MMR homologous genes), and Table S4 (primers for microsatellite associated genes), respectively. Real-time PCR was performed using an ABI PRISM® 7700 Sequence Detection System (Applied Biosystems, Foster City, USA) and SYBR Premix Ex Taq (TaKaRa, Tokyo, Japan) as a DNA-specific fluorescent dye. Thermal cycling conditions consisted of an initial denaturation step at 95°C for 1 min, followed by 40 cycles of 15 s at 95°C and 1 min at 60°C. All the PCR reactions were repeated for at least three times. The Ct (threshold cycle) was defined as the number of cycles required for the fluorescence signal to exceed the detection threshold. Data were analyzed by using the software provided by ABI Company (Applied Biosystems, Foster City, USA) and calculated by the 2^–ΔΔCt^ method. Quantitative results were given as means ± SD.

## Supporting Information

Figure S1 **Evaluation of the relative transcript levels of the three putative microsatellite-regulated genes in two sets of rice genotypes by qRT-PCR.**
(PPT)

Table S1 **Information for the 96 microsatellite markers used in this study.**
(XLS)

Table S2 **The accession numbers and the amplified sequences of the 30 sequenced loci in this study.**
(XLS)

Table S3 **Oligonucleotide primer sets used for detecting the transcript of rice** ***β-actin*** **and MMR genes.**
(DOC)

Table S4 **Oligonucleotide primer sets used for detecting the transcript of microsatellite associated genes.**
(XLS)
