# Correction: Extensive Microsatellite Variation in Rice Induced by Introgression from Wild Rice (*Zizania latifolia* Griseb.)

**DOI:** 10.1371/annotation/1506bbde-ee47-49eb-a31e-6c7ff5fff0f9

**Published:** 2013-09-13

**Authors:** Zhenying Dong, Hongyan Wang, Yuzhu Dong, Yongming Wang, Wei Liu, Gaojian Miao, Xiuyun Lin, Daqing Wang, Bao Liu

A third grant from the National Natural Science Foundation of China was incorrectly omitted from the Funding Statement. The number of this grant is: 31100172. 

