# Supplementary material for: Extensive Microsatellite Variation in Rice Induced by Introgression from Wild Rice (Zizania latifolia Griseb.)
Source: PLoS One. 2013 Apr 24;8(4):e62317. doi: 10.1371/journal.pone.0062317 (PMC3634730; doi:10.1371/journal.pone.0062317)
Supplement: Figure S1 — Evaluation of the relative transcript levels of the three putative microsatellite-regulated genes in two sets of rice genotypes by qRT-PCR. (PPT) [file pone.0062317.s001.ppt]

## Slide 1
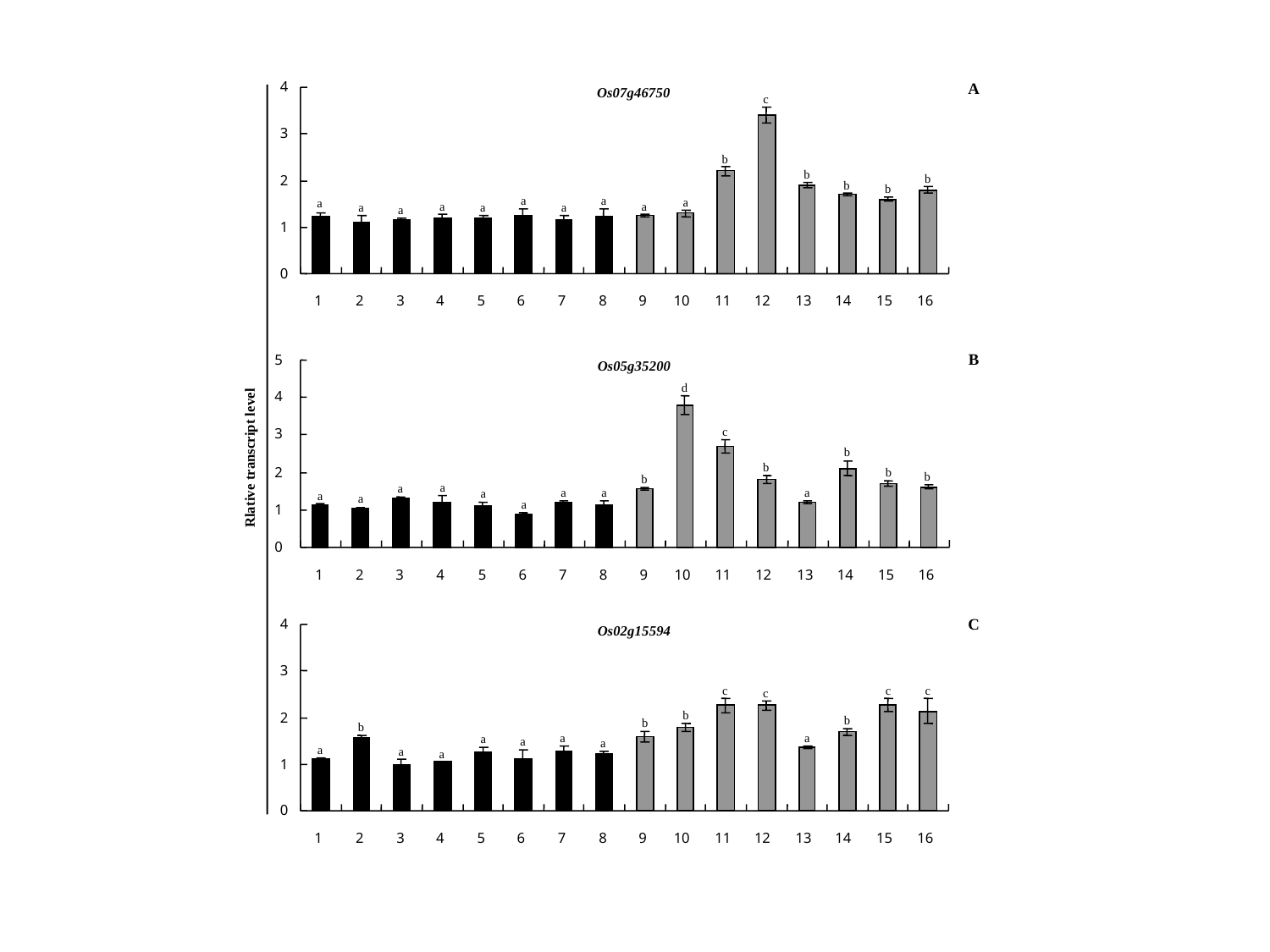

A
Os07g46750
4
c
3
b
b
b
b
2
b
a
a
a
a
a
a
a
a
a
a
1
0
1
2
3
4
5
6
7
8
9
10
11
12
13
14
15
16
B
Os05g35200
5
d
4
c
3
b
Rlative transcript level
b
b
b
2
b
a
a
a
a
a
a
a
a
a
1
0
1
2
3
4
5
6
7
8
9
10
11
12
13
14
15
16
C
4
Os02g15594
3
c
c
c
c
b
b
b
2
b
a
a
a
a
a
a
a
a
1
0
1
2
3
4
5
6
7
8
9
10
11
12
13
14
15
16

## Slide 2
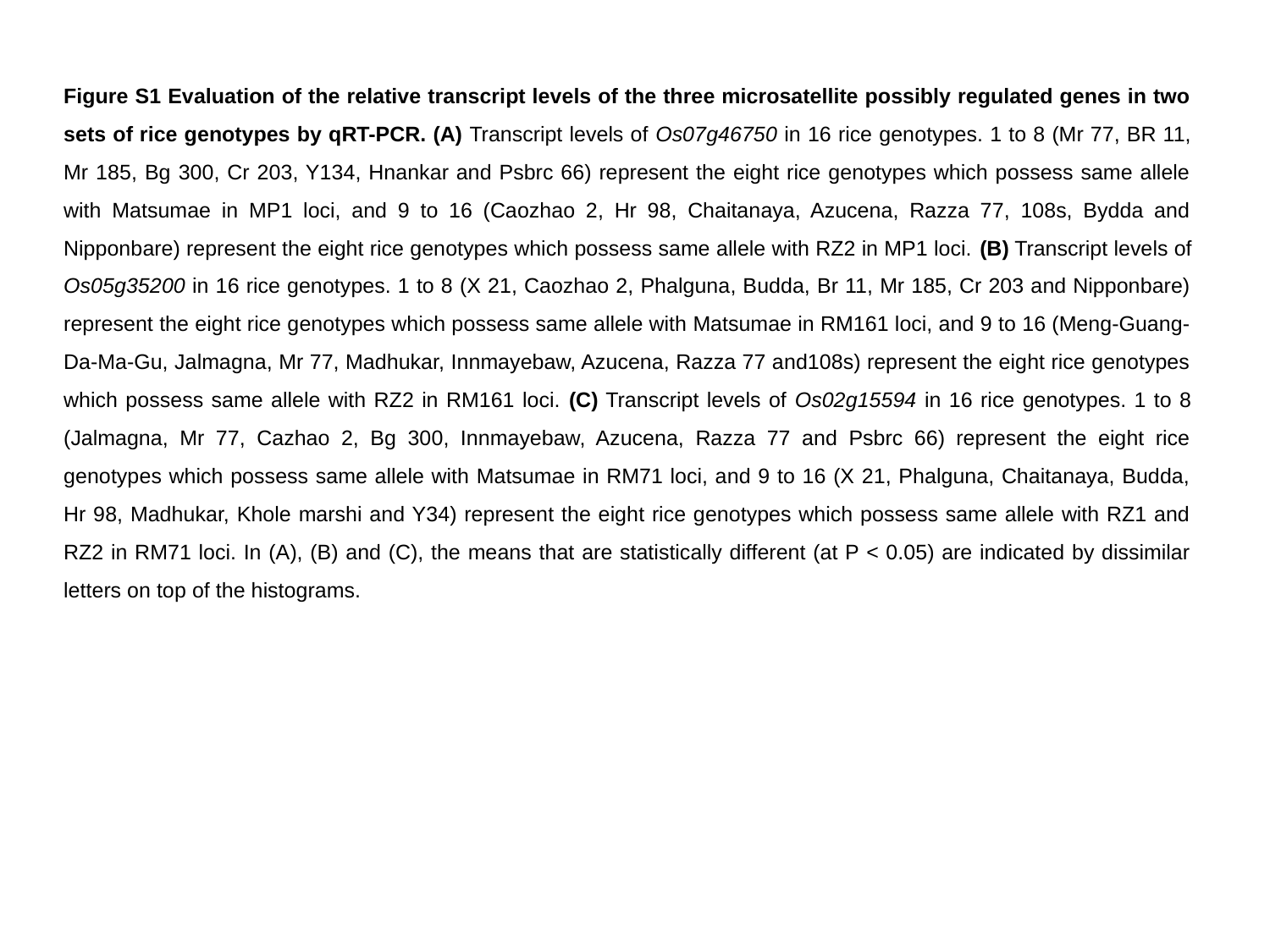

Figure S1 Evaluation of the relative transcript levels of the three microsatellite possibly regulated genes in two sets of rice genotypes by qRT-PCR. (A) Transcript levels of Os07g46750 in 16 rice genotypes. 1 to 8 (Mr 77, BR 11, Mr 185, Bg 300, Cr 203, Y134, Hnankar and Psbrc 66) represent the eight rice genotypes which possess same allele with Matsumae in MP1 loci, and 9 to 16 (Caozhao 2, Hr 98, Chaitanaya, Azucena, Razza 77, 108s, Bydda and Nipponbare) represent the eight rice genotypes which possess same allele with RZ2 in MP1 loci. (B) Transcript levels of Os05g35200 in 16 rice genotypes. 1 to 8 (X 21, Caozhao 2, Phalguna, Budda, Br 11, Mr 185, Cr 203 and Nipponbare) represent the eight rice genotypes which possess same allele with Matsumae in RM161 loci, and 9 to 16 (Meng-Guang-Da-Ma-Gu, Jalmagna, Mr 77, Madhukar, Innmayebaw, Azucena, Razza 77 and108s) represent the eight rice genotypes which possess same allele with RZ2 in RM161 loci. (C) Transcript levels of Os02g15594 in 16 rice genotypes. 1 to 8 (Jalmagna, Mr 77, Cazhao 2, Bg 300, Innmayebaw, Azucena, Razza 77 and Psbrc 66) represent the eight rice genotypes which possess same allele with Matsumae in RM71 loci, and 9 to 16 (X 21, Phalguna, Chaitanaya, Budda, Hr 98, Madhukar, Khole marshi and Y34) represent the eight rice genotypes which possess same allele with RZ1 and RZ2 in RM71 loci. In (A), (B) and (C), the means that are statistically different (at P < 0.05) are indicated by dissimilar letters on top of the histograms.
