# Supplementary material for: Extensive Microsatellite Variation in Rice Induced by Introgression from Wild Rice (Zizania latifolia Griseb.)
Source: PLoS One. 2013 Apr 24;8(4):e62317. doi: 10.1371/journal.pone.0062317 (PMC3634730; doi:10.1371/journal.pone.0062317)
Supplement: Table S3 — Oligonucleotide primer sets used for detecting the transcript of rice β-actin and MMR genes. (DOC) [file pone.0062317.s004.doc]

**Table S3** Oligonucleotide primer sets used for detecting the transcript of rice *β-actin* and MMR genes

| Forward primer (5′-3′) | Reverse primer (5′-3′) | Specificity |
| --- | --- | --- |
| TGGCAGGTCCAGCATTTA | CCTTCGATCTGTGAGCACTA | *MSH1* |
| TCGGCGGGATTATTACACT | TTGCGGGCGATGGTCTC | *MSH2* |
| GATGGAGATGAACGAGGAGAA | AACCCTTGAGATAGCGAACAC | *MSH3* |
| GACTGCTTGATGTTGCTC | TAATGTCCTTCTGGGGTA | *MSH4* |
| TTGCTGGTGTTCCGAGTG | TGGTATTGTTGGTCCTTTGC | *MSH5* |
| CACAAATCAGTTCTGCCTAT | TGAGCCCTGAGCATGTAA | *MSH6* |
| AATGGATCGGATTATGTCTGG | TGGTAGTGAGTTGCGAAGAGC | *MSH7* |
| TCCAATGATGACTACCCC | AAGCCCCATAGACACTCC | *MLH1* |
| CGTCTGCGATAATGGAACTG | TCTGGGTCATCTTCTCACGA | *β-actin* |
